# Use of UV-C radiation to disinfect non-critical patient care items: a laboratory assessment of the Nanoclave Cabinet

**DOI:** 10.1186/1471-2334-12-174

**Published:** 2012-08-03

**Authors:** Ginny Moore, Shanom Ali, Elaine A Cloutman-Green, Christina R Bradley, Martyn AC Wilkinson, John C Hartley, Adam P Fraise, A Peter R Wilson

**Affiliations:** 1 Clinical Microbiology and Virology, University College London Hospitals NHS Foundation Trust, London, UK; 2 Great Ormond Street Hospital for Sick Children, London, UK; 3 Hospital Infection Research Laboratory, Queen Elizabeth Hospital, Birmingham, UK; 4 Department of Microbiology, UCLH Environmental Research Group, Royal Free Hampstead NHS Trust, London, NW3 2QG, UK

**Keywords:** Ultraviolet radiation, Surface disinfection, Nosocomial pathogens, Adenovirus

## Abstract

**Background:**

The near-patient environment is often heavily contaminated, yet the decontamination of near-patient surfaces and equipment is often poor. The Nanoclave Cabinet produces large amounts of ultraviolet-C (UV-C) radiation (53 W/m^2^) and is designed to rapidly disinfect individual items of clinical equipment. Controlled laboratory studies were conducted to assess its ability to eradicate a range of potential pathogens including *Clostridium difficile* spores and Adenovirus from different types of surface.

**Methods:**

Each test surface was inoculated with known levels of vegetative bacteria (10^6^ cfu/cm^2^), *C. difficile* spores (10^2^-10^6^ cfu/cm^2^) or Adenovirus (10^9^ viral genomes), placed in the Nanoclave Cabinet and exposed for up to 6 minutes to the UV-C light source. Survival of bacterial contaminants was determined via conventional cultivation techniques. Degradation of viral DNA was determined via PCR. Results were compared to the number of colonies or level of DNA recovered from non-exposed control surfaces. Experiments were repeated to incorporate organic soils and to compare the efficacy of the Nanoclave Cabinet to that of antimicrobial wipes.

**Results:**

After exposing 8 common non-critical patient care items to two 30-second UV-C irradiation cycles, bacterial numbers on 40 of 51 target sites were consistently reduced to below detectable levels (≥ 4.7 log_10_ reduction). Bacterial load was reduced but still persisted on other sites. Objects that proved difficult to disinfect using the Nanoclave Cabinet (e.g. blood pressure cuff) were also difficult to disinfect using antimicrobial wipes. The efficacy of the Nanoclave Cabinet was not affected by the presence of organic soils. *Clostridium difficile* spores were more resistant to UV-C irradiation than vegetative bacteria. However, two 60-second irradiation cycles were sufficient to reduce the number of surface-associated spores from 10^3^ cfu/cm^2^ to below detectable levels. A 3 log_10_ reduction in detectable Adenovirus DNA was achieved within 3 minutes; after 6 minutes, viral DNA was undetectable.

**Conclusion:**

The results of this study suggest that the Nanoclave Cabinet can provide rapid and effective disinfection of some patient-related equipment. However, laboratory studies do not necessarily replicate ‘in-use’ conditions and further tests are required to assess the usability, acceptability and relative performance of the Nanoclave Cabinet when used *in situ*.

## Background

Important nosocomial pathogens such as methicillin-resistant *Staphylococcus aureusClostridium difficile* and vancomycin-resistant enterococci are often present on inanimate surfaces within the local environment of infected patients [1-3]. Many of these surfaces (e.g. blood-pressure cuffs, bed rails, bedside furniture) only come into contact with a patient’s intact skin – a highly effective barrier against microbes. Consequently, such surfaces are considered “non-critical” and rather than being returned to a central sterilising services department for re-processing, can be decontaminated *in situ*[4,5].

Routine cleaning of the near-patient environment has been associated with a reduction in surface contamination [2]. However, cleaning of near-bedside equipment and furniture is not always adequate, especially if it is a nursing responsibility and they are busy [5,6]. Inadequate cleaning allows microbial contaminants to survive and persist on environmental surfaces and whilst non-critical surfaces pose little direct risk to patients [4], they can act as a source from which healthcare workers can contaminate their hands and may serve as vectors for cross-transmission.

Ultraviolet-C (UV-C) radiation has been used for many years to disinfect water and its bactericidal effects, due mainly to its inactivation of microbial DNA, have been well documented [7]. More recently, UV-C has been used to disinfect hospital rooms [8-10] and its ability to reduce the number of healthcare–associated pathogens within the near-patient environment has been demonstrated [9-11]. However, for microorganisms to be destroyed they must be directly exposed to UV-C irradiation; any surface not in the direct path of the UV-C rays will not be effectively disinfected [8].

The Nanoclave Cabinet (Nanoclave Technologies LLP, London, UK) produces large amounts of UV-C light. Its purpose is to rapidly disinfect clinical equipment, furniture and electronic devices. Inside the Cabinet are 48 UV-C lamps (32 × 30 W and 16 × 25 W) mounted, in banks of eight, to each of the six internal surfaces, including the door. Angled mirrored reflectors help minimise shadowing by directing and concentrating the UV-C rays onto the item to be disinfected. This six-sided emission of UV-C light means any item placed in the cabinet is subjected to a dosage of 53 W/m^2^.

The aim of this study was to assess, under controlled laboratory conditions, the ability of the Nanoclave Cabinet to effectively disinfect a range of artificially contaminated non-critical patient care items.

## Methods

### The Nanoclave Cabinet

**Figure 1:**
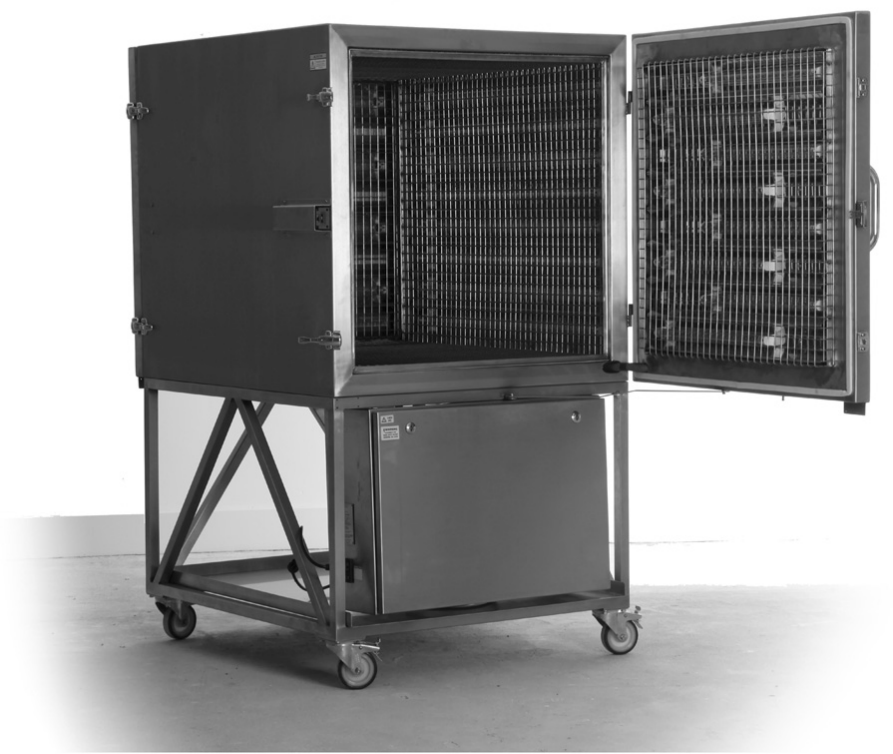
The Nanoclave Cabinet.

The Nanoclave Cabinet is made from stainless steel and can be manufactured in a range of different dimensions. The Cabinet supplied for use during this investigation measured 129 cm × 94 cm × 89 cm (l × w × h) and was mounted on a base which raised the height of the unit to 1.6 m (Figure 1). To ensure that all the UV-C lamps were working correctly, a device controller measured the power consumption of the lamps during operation. Any significant drop in power resulted in the failure and cessation of the cycle. The Nanoclave Cabinet also incorporates a data logging feature which, for additional safety, is independent from the device controller. Current meters monitor the current drawn by each bank of lamps and UV-C sensors monitor the actual UV-C output of the lamps. These data are collected onto an SD card and can be printed using a thermal printer.

### Effectiveness of the Nanoclave Cabinet against a range of pathogenic bacteria

#### Test organisms

Testing involved a range of potential nosocomial pathogens: methicillin-sensitive *Staphylococcus aureus* (MSSA; NCTC 10788), methicillin-resistant *Staphylococcus aureus* (MRSA; EMRSA-15 variant B1 (environmental isolate)), *Enterococcus hirae* (NCTC 12367), vancomycin-resistant *Enterococcus faecalis* (VRE; clinical isolate), *Escherichia coli* (NCTC 10418), multi-resistant *Acinetobacter baumannii* (MRAB; OXA-23 clone 1 (clinical isolate)), extended spectrum beta-lactamase (ESBL) producing *Klebsiella pneumoniae* (environmental isolate) and *Pseudomonas aeruginosa* (NCTC 6749).

Clinical isolates were taken from clinical specimens and stored in the microbiology laboratory. Only the isolated microorganisms and not the specimens (e.g. urine; sputum; faecal samples) were stored. Clinical isolates were fully anonymised and testing was only to assess the effectiveness of the Nanoclave Cabinet. The organisms were not tested to reveal any additional information and there was no way to link them to individual patients. Thus, ethics consideration was not deemed necessary by UCLH Research and Development.

#### Ability of the Nanoclave Cabinet to disinfect non-critical patient care items

##### Preparation of test items

Eight items of near-bedside equipment of the type and in the condition of those likely to be found in the ward environment were included in the study; a blood pressure gauge, a patient call button, an infusion pump, a tympanic thermometer, an oximeter base unit, a computer keyboard (and mouse), a TV remote control and a blood pressure cuff. Each surface was marked with up to nine individual sample points.

Prior to each experiment, each test surface was cleaned using a (hand hot) damp microfibre cloth, left to air-dry under ambient conditions and disinfected using 70% alcohol spray. The efficacy of this cleaning protocol was assessed using agar contact plates and residual microbial numbers were consistently reduced to below detectable levels.

##### Exposure of test items to UV-C radiation

A single colony of MRSA, VRE, MRAB or *Kleb pneumoniae* was aseptically transferred into 10 ml sterile nutrient broth (Oxoid, Basingstoke, UK). A stationary-phase culture (~10^8^ cfu/ml) was obtained by incubating the bacteria at 37°C for 18 h. After incubation, the culture was transferred to a sterile universal container and centrifuged at 1500 × g for 10 min. The supernatant was discarded and the pellet re-suspended in 10 ml sterile ¼-strength Ringer’s solution (an isotonic salt solution; Oxoid).

For each test item, 10 μl of bacterial suspension (containing approximately 10^6^ cfu) was inoculated onto each sample point and, rather than being left as a droplet, spread over a 1 cm^2^ test area. Immediately after inoculation, the test item was placed in the Nanoclave Cabinet on a stainless steel lattice rack and exposed for 30 sec to the UV-C light source.

Although the base of the item (i.e. the surface facing the rack) was exposed to UV-C light emitted from the base of the cabinet, any sample point in direct contact with the lattice bars remained protected from the rays. Thus, after irradiation, to ensure the entire surface area was exposed to a UV-C dose of at least 1,590 J/m^2^ (53 W/m^2^ × 30 sec exposure), the positioning of the object within the Cabinet was altered and the irradiation cycle repeated.

After exposure, a pre-moistened cotton-tipped swab was used to sample each sample point. Each swab was placed in 1 ml ¼-strength Ringer’s solution and vortexed to release the bacteria. One hundred microlitres of the resulting suspension was plated onto a pre-poured blood agar plate (Oxoid) and incubated at 37°C for 24 hours.

##### Non-exposed control samples

Test items were inoculated as previously described. Immediately after inoculation, each test area was sampled using a pre-moistened cotton-tipped swab. Each swab was placed in 9 ml ¼-strength Ringer’s solution and vortexed to release the bacteria. The resulting suspension was diluted 100-fold and 100 μl of the diluted sample plated onto blood agar. Plates were incubated at 37°C for 24 hours.

#### Comparative performance of antimicrobial wipes

To compare the efficacy of the Nanoclave Cabinet with that of an antimicrobial wipe, three patient care items (blood pressure cuff, tympanic thermometer, patient call button) were inoculated with a representative organism (*Acinetobacter baumannii*). Selected test areas were cleaned ‘poorly’ (one wiping stroke), ‘moderately well’ (two wipes) or ‘thoroughly’ (four wipes) using an antimicrobial wipe (VWR International Disinfectant Wipes: active ingredients: peroxides, benzalkonium chloride; VWR International, Lutterworth, UK). Each test area was sampled with a pre-moistened swab which, prior to plating, was vortexed within 1 ml of neutralising solution (phosphate buffered saline incorporating 3% Tween 80 (w/v), 0.3% lecithin (w/v), 0.1% sodium thiosulphate (w/v)).

#### Effect of organic soiling on the efficacy of the Nanoclave Cabinet

A stationary-phase culture of MSSA, *E. hirae, E. coli* or *P. aeruginosa* was prepared as previously described. After centrifugation, cell pellets were re-suspended in either 0.03% bovine serum albumin (BSA; w/v) sterilized by membrane filtration or, to represent heavy soiling, 0.3% BSA (w/v) and 0.3% “packed” sheep erythrocytes (v/v), which were prepared as follows. Three millilitres of sterile defribinated sheep blood (TCS Biosciences Ltd, Buckingham, UK) was centrifuged at 800 × g for 10 minutes. The supernatant was discarded and the pellet re-suspended in a balanced salt solution. This process was repeated until the supernatant was colourless. The packed erythrocytes were then re-suspended and added to a sterile solution of BSA (3.0 g bovine albumin (fraction V), 0.1 g tryptone, 0.85 g sodium chloride, 97 ml distilled water). The resulting suspension was diluted 10-fold.

Sterile stainless steel discs (1 cm in diameter) were inoculated with 20 μl bacterial suspension (~ 10^6^ cfu) and allowed to dry for 80 minutes at 30°C. Two discs were then attached to each surface of a plastic cube, placed in the Nanoclave Cabinet and exposed for 60 sec to the UV-C light source. Thus, each of 12 discs was positioned either vertically or horizontally and exposed to a UV-C dose of 3,180 J/m^2^. After exposure, each disc was aseptically transferred to 10 ml tryptone soya broth containing sterile glass beads and vortexed for 1 min. The resulting suspension was diluted 10-fold and 100 μl of the diluted sample plated onto tryptone soya agar. Control discs were inoculated and incubated as described but were cultured without having been exposed to UV-C. All plates were incubated at 37°C for 24 hours.

#### Analysis of results

For each test surface, the number of colonies recovered from each irradiated or wiped test area was subtracted from the number of colonies recovered from the corresponding control sample. The results were used to calculate the mean log reduction in microbial viability and, thus, the efficacy of the Nanoclave Cabinet or antimicrobial wipes. Data analysis was performed using Microsoft Excel 2007. Statistical significance was determined by use of t tests and was at a level of *P* < 0.05.

### Effectiveness of the Nanoclave Cabinet against *Clostridium difficile* spores

Spore suspensions of *Clostridium difficile* were prepared as previously described [12] and stored in a 1:1 solution of alcohol (70%) and phosphate buffered saline.

A stainless steel sheet was cleaned using a (hand hot) damp microfibre cloth, left to air-dry under ambient conditions and disinfected using 70% alcohol spray. A spore suspension of *C. difficile* 027 (clinical isolate) was centrifuged at 1500 × g for 10 min and re-suspended in 10 ml sterile ¼-strength Ringer’s solution. A 10 μl aliquot (containing approximately 10^6^ cfu) was inoculated onto the steel surface and spread over a 1 cm^2^ test area. Immediately after inoculation, the sheet was placed in the Nanoclave Cabinet and exposed to two 60 sec UV-C cycles. After exposure, the test surface was sampled using a pre-moistened cotton-tipped swab which was transferred to 1 ml ¼-strength Ringer’s solution and vortexed to release the spores. One hundred microlitres of the resulting suspension was plated onto a pre-poured Brazier’s agar plate (Oxoid) and incubated under anaerobic conditions at 37°C for 48 hours. Experiments comprised a minimum of three replicate samples and were repeated to incorporate lower inoculum levels and longer exposure times. The effect of organic soiling was investigated by re-suspending spores of *C. difficile* NCTC 11209 (ribotype 001) in 0.03% BSA and inoculating sterile stainless steel discs as described previously.

### Effectiveness of the Nanoclave Cabinet against Adenovirus

A stainless steel sheet and a ceramic tile were cleaned and disinfected as previously described. Adenovirus species (serotype 31) was grown in a Vero cell line. A 10 μl aliquot (containing approximately 10^9^ viral genomes) was inoculated onto the test surface and spread over a 5 cm^2^ test area. After being allowed to air-dry (ambient conditions) for 2 h, the sheet (or tile) was placed in the Nanoclave Cabinet and exposed to two 30 sec UV-C cycles. After exposure, the test surface was sampled using a pre-moistened cotton-tipped swab which was transferred to 0.5 ml molecular grade water and vortexed to release the virus particles. Viral nucleic acid was extracted from 200 μl of the resulting suspension using a DNA Miniprep Kit (Qiagen, Crawley, UK) and eluted into 100 μl UV irradiated buffer. Ten microlitres of the extract was processed using a semi-quantitative Adenovirus real time polymerase chain reaction (PCR) [13]. All PCR’s were run with a negative extraction, as well as negative and positive controls; the latter to monitor assay performance across runs. Experiments comprised four replicate samples and were repeated to incorporate longer exposure times.

## Results

### Ability of the Nanoclave Cabinet to disinfect non-critical patient care items

#### Effectiveness of the Nanoclave Cabinet against vegetative bacteria

Fifty-one individual sample sites associated with eight near-bedside items of clinical equipment and furniture were inoculated with MRSA, VRE, MRAB and *Klebsiella pneumoniae*. Loss in microbial viability varied depending on surface type (Table 1) but exposing 40 of the 51 target sites (78%) to two 30-second UV-C irradiation cycles consistently reduced the number of contaminating organisms by at least 4.7 log_10_ values and/or to below detectable levels (10 cfu).

**Table 1:** Ability of the Nanoclave Cabinet to disinfect non-critical patient care items

	**minimum and maximum log**_**10**_**reduction after exposure to two 30-second UV-C cycles**^***a***^															
	**Blood pressure gauge (n = 8)**		**Patient call button (n = 8)**		**Infusion pump (n = 5)**		**Tympanic thermometer (n = 9)**		**Oximeter (base unit) (n = 7)**		**Computer keyboard/mouse (n = 4)**		**TV remote control (n = 4)**		**Blood pressure cuff (n = 6)**	
Pathogen ^*b*^	min	max	min	max	min	max	min	max	min	max	min	max	min	max	min	max
MRSA	4.40	>5.29	>4.74	>5.17	>4.94	>5.08	2.16	>5.45	>5.25	>5.48	>4.97	>5.10	>5.05	>5.32	1.93	>5.13
VRE	>5.11	>5.23	>5.05	>5.21	>4.93	>5.30	1.49	>5.44	>4.91	5.27	4.28	>5.03	4.93	>5.16	2.13	>5.00
*A. baumannii*	3.44	>5.54	5.32	>5.59	>5.39	>5.56	2.29	>5.64	>5.13	>5.48	4.90	>5.74	>5.33	>5.75	3.46	>5.39
*Kleb. pneum*	2.76	>5.19	>4.84	>5.25	4.04	5.07	1.02	>5.11	>4.33	>5.08	>5.11	>5.21	>5.05	>5.12	3.24	>5.34

^*a*^initial inoculum: 10^6^ cfu/cm^2^.

^*b*^test organism suspended in ¼-strength Ringer’s solution.

The Nanoclave Cabinet was less effective when used to disinfect the tympanic thermometer and the blood pressure cuff (Table 1). Although two 30-second UV-C cycles reduced bacterial numbers on some sites to below detectable levels, on others, bacterial numbers were reduced by less than 2 log_10_ values.

#### Comparative performance of the Nanoclave Cabinet and antimicrobial wipes

‘Thoroughly’ cleaning the tympanic thermometer with an antimicrobial wipe (four wiping strokes) reduced the number of bacteria on most sample points to below detectable levels (Table 2). A single wiping motion (defined as a ‘poor’ clean) was less effective than the Nanoclave Cabinet in reducing contamination levels on the display panel but more effective when used to disinfect the probe receptor and earpiece holder. When used to disinfect the infra-red sensor neither antimicrobial wipes nor the Nanoclave Cabinet were particularly effective in reducing bacterial numbers. Whilst two 30-second UV-C cycles achieved a 2.30 log_10_ reduction, cleaning with an antimicrobial wipe only reduced bacterial numbers by 2.14 log_10_ values (Table 2).

**Table 2:** Comparative performance of the Nanoclave Cabinet and antimicrobial wipes when used to disinfect patient care items

	**Mean (± SD) log**_**10**_**reduction**^***a***^**(n = 3)**			
	**Nanoclave**	**Antimicrobial wipes**		
	**(2 × 30 sec)**	**‘poor’ wiping**	**‘moderate' wiping**	**‘thorough’ wiping**
**Tympanic thermometer**				
display panel	>5.49	4.45 ± 1.03	5.04 ± 0.60	>5.39
infra-red sensor	2.29 ± 0.93	1.94 ± 0.03	1.96 ± 0.25	2.14 ± 0.14
plastic lid	>5.64	>5.40	>5.40	>5.40
probe receptor	4.55 ± 1.07	5.16 ± 0.17	>5.48	>5.48
earpiece holder	3.44 ± 0.13	5.25 ± 0.40	5.28 ± 0.35	>5.49
**Blood pressure cuff**				
Velcro (hook)	3.60 ± 0.98	1.91 ± 0.07	2.42 ± 0.14	2.66 ± 0.09
Velcro (loop)	4.28 ± 0.96	1.50 ± 0.03	2.26 ± 0.19	2.67 ± 0.06
inner cuff surface	3.46 ± 1.47	1.73 ± 0.09	2.65 ± 0.08	2.30 ± 0.08
pump	>5.39	2.38 ± 0.15	2.90 ± 0.08	>5.60
pump tubing	>5.07	2.75 ± 0.40	3.94 ± 1.22	>5.33

^*a*^ initial inoculum: 10^6^ cfu/cm^2^.

When used to disinfect a blood pressure cuff, the Nanoclave cabinet reduced the number of bacteria on the pump and pump tubing by more than 5 log_10_ values (Table 2). ‘Thorough’ cleaning using an antimicrobial wipe achieved a similar log reduction but less effective wiping reduced bacterial numbers by between 2.38 and 3.94 log_10_ values. Antimicrobial wipes were least effective when used to disinfect the inner cuff surface and either side of the velcro fastening. The Nanoclave Cabinet was comparatively more effective and reduced the number of bacteria contaminating these surfaces by between 3.46 and 4.28 log_10_ values (Table 2).

When used to disinfect the patient call button, the Nanoclave Cabinet reduced the number of bacteria on all target sites to below detectable levels (> 5.3 log_10_ values). Cleaning using an antimicrobial wipe was equally effective although a ‘poor’ wiping technique allowed organisms to persist on the rear panel and rubber grip.

#### Effect of organic soiling on the efficacy of the Nanoclave Cabinet

In the presence of low level soiling (0.03% BSA), two 30-second UV-C irradiation cycles reduced MSSA, *Enterococcus hirae*, *Escherichia coli* and *Pseudomonas aeruginosa* numbers to below detectable levels and achieved at least a 5.8 log_10_ reduction in microbial viability. Increasing the organic challenge had little effect upon the efficacy of the Nanoclave Cabinet which, in the presence of BSA (0.3%) and red blood cells reduced MSSA and *P. aeruginosa* numbers to below detectable levels (i.e. achieved a 6 log_10_ reduction) within 60 seconds (Table 3).

**Table 3:** Effect of organic soiling on the efficacy of the Nanoclave Cabinet

	**Mean (± SD) log**_**10**_**reduction**	
	**Light soiling (n = 36) 0.03% BSA**	**Heavy soiling (n = 12) 0.3% BSA + 0.3% sheep erythrocytes**
MSSA	> 7.18	6.19 ± 0.76
*P. aeruginosa*	> 6.12	>5.99
*E. coli*	> 5.84	not tested
*E. hirae*	> 6.15	not tested
*C. difficile* spores (ribotype 001)	3.55 ± 0.47	not tested

#### Effectiveness of the Nanoclave Cabinet against Clostridium difficile spores

**Figure 2:**
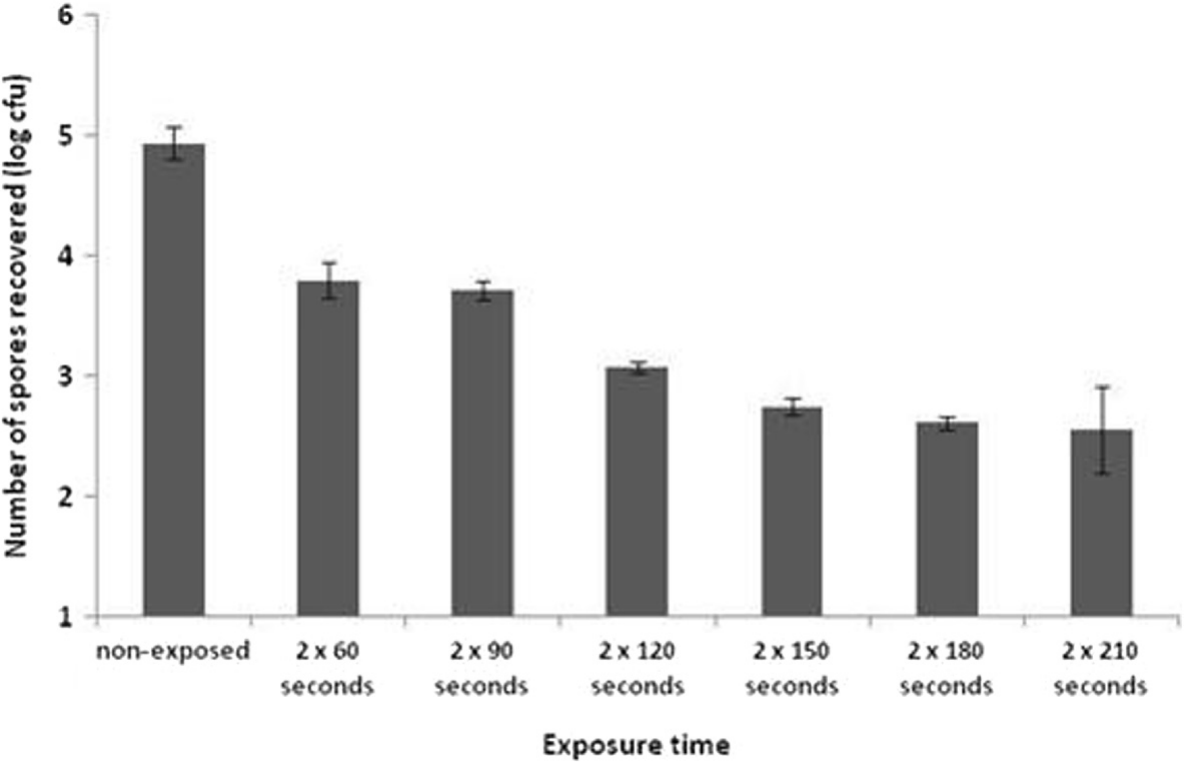
**The effect of cycle duration upon the mean number of*Clostridium difficile*027 spores recovered from a stainless steel surface (n = 5; error bars indicate the standard deviation).**

**Figure 3:**
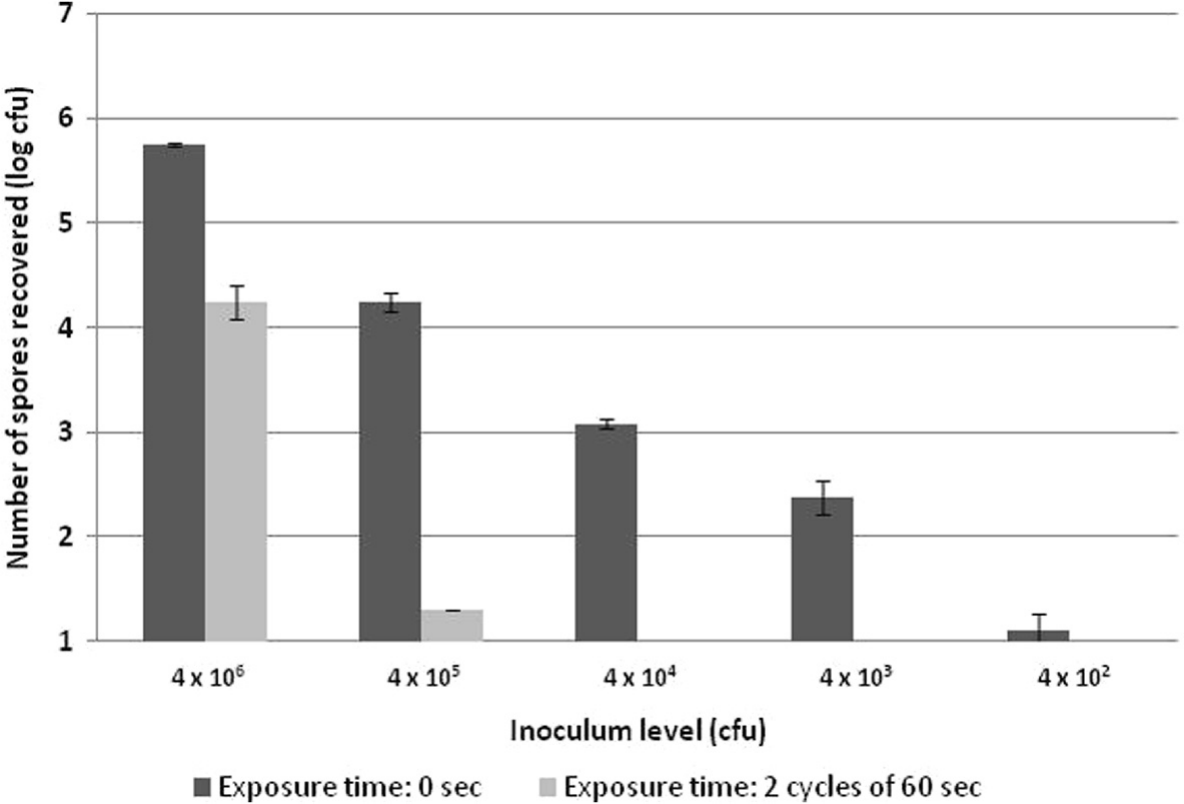
**Efficacy of the Nanoclave Cabinet against the spores of*Clostridium difficile*ribotype 027: the effect of inoculum level (n = 3; error bars indicate the standard deviation).**

The Nanoclave Cabinet was less effective against *C. difficile* spores, particularly those of the clinical strain. Two 30-second cycles achieved a 3.55 log_10_ reduction in *C. difficile* NCTC 11209 spore numbers (Table 3). In comparison, two 60-second cycles reduced the number of 027 spores by just 1.14 log_10_ values (Figure 2). A 2.18 log_10_ reduction was achieved after a total exposure time of 5 min (i.e. two 150-second cycles). Increasing the cycle time further had no significant effect (*P* > 0.05; Figure 2). However, when the initial inoculum was ≤ 10^4^ cfu/cm^2^ two 60-second UV cycles were sufficient to reduce the number of *C. difficile* 027 spores to below detectable levels (Figure 3).

#### Effectiveness of the Nanoclave Cabinet against Adenovirus species A

Viability assays were not available for Adenovirus, so persistence of viral DNA following inoculation of viable cell culture, detected by polymerase chain reaction (PCR), was used as a surrogate marker. PCR may detect both viable and non-viable virus, depending upon the integrity of the DNA present on the surface. UV-C degrades DNA, so there will be loss of viability before total loss of detectable DNA by PCR, but the point at which all viable virus is lost is not known. The levels of retrievable viral genomes are recorded as a function of the PCR assay using the Cycle Threshold (CT) values. The CT is the number of doubling cycles required before the assay became positive. A small CT represents a higher starting load and each 3.3 CT increase between samples equates to a 1 log_10_ reduction in detectable viral genome. A CT value of 45 is the assay end-point and DNA considered ‘undetectable’.

The ability of the Nanoclave Cabinet to degrade Adenovirus DNA was only tested on smooth metal or ceramic surfaces. On these surfaces the UV-C is shown to degrade Adenovirus DNA by the increase in CT value following successive exposures (Table 4). Regardless of test surface, six 30-second cycles (3 minutes) increased the mean CT between 9 and 10 CT values. Thus, a total exposure time of 3 minutes resulted in a 3 log_10_ reduction in detectable viral DNA. After an exposure time of 6 minutes, viral DNA was undetectable on both the stainless steel and ceramic test surface (i.e. a 6 log_10_ reduction had been achieved).

**Table 4:** Effect of cycle duration upon the degradation of Adenovirus DNA

**Exposure time**	**Mean Cycle Threshold (CT) value**^***a***^**(n = 4)**	
	**Stainless steel sheet**	**Ceramic tile**
0 min (control)	17	18
1 min	22	22
2 min	25	27
3 min	27	27
4 min	33	34
5 min	31	45
6 min	45	45

^*a*^A 3.3 CT increase equates to a 1 log reduction in detectable viral genome. A CT value of 45 is the assay end-point and when DNA is considered undetectable.

## Discussion

The routine cleaning and disinfection of the near-patient environment is often inadequate and many items of near-patient equipment and furniture have been identified as potential bacterial reservoirs [3,6,14-18]. The efficacy of many traditionally used products and practices has been questioned as has their human and ecological safety [19]. Such concerns have prompted an increasing interest in the use of additional or alternative surface disinfectants, for example, self-disinfecting surfaces [20], hydrogen peroxide vapour [21] and ultraviolet light.

Ultraviolet irradiation is considered an acceptable and environmentally friendly means of disinfecting surfaces in healthcare settings [10]. The Tru-D Rapid Room Decontamination device (Lumalier Corporation) can eliminate vegetative bacteria and *C. difficile* spores from contaminated surfaces within 15 min and 50 min respectively [9,10]. However, such UV-C devices cannot be used when the room is occupied and a lengthy cycle time is impractical if a rapid turn-over of beds is required.

The Nanoclave Cabinet is used to disinfect individual patient-care items. Any item placed in the Cabinet is subjected to six-sided emission of UV-C light both directly and via angled mirror reflectors. During the current study, the Nanoclave Cabinet was used to disinfect a variety of non-critical patient care items and the UV-C light caused no observable damage. However, the range of surface materials tested was by no means exhaustive. Not all materials are suitable. The Medicines and Healthcare products Regulatory Agency (MHRA) has advised that the outer coating of flexible endoscopes may be damaged by direct exposure to ultraviolet light [22]. UV-C light is also injurious to soft contact lens polymers albeit at a dose much higher (250 mW/cm^2^) than that generated by the Nanoclave Cabinet (5.3 mW/cm^2^) [23].

Each patient-care item was irradiated for 30 sec. The surface was rotated to expose those areas initially in contact with the rack, and the irradiation cycle repeated. Two irradiation cycles ensured the entire surface area was exposed to a UV-C dose of 1,590 J/m^2^ (53 W/m^2^ × 30 s exposure time) and that much of the surface was subjected to twice this dose (3,180 J/m^2^; 53 W/m^2^ × 60 s exposure time). This was sufficient to reduce the number of vegetative contaminants on the majority of sample points by at least 4.7 log_10_ values. However, not all test points demonstrated the same reduction. Poor penetration of the UV-C rays and/or significant shadowing, enabled bacteria to persist on the tympanic thermometer and the blood pressure cuff (Table 2). It was possible to reach the deep recesses associated with the thermometer (e.g. probe receptor; earpiece holder) with an antimicrobial wipe and wiping reduced the number of contaminating organisms to below detectable levels. In contrast, although surface contamination decreased as the thoroughness of wiping increased, antimicrobial wipes were less effective than the Nanoclave Cabinet in disinfecting the blood pressure cuff, particularly the Velcro fastener.

The Nanoclave Cabinet was less effective on surfaces contaminated with *C. difficile* spores. Previous studies have also found *C. difficile* spores to be more resistant to UV-C radiation than vegetative bacteria [9,10]. Exposing a highly contaminated surface (10^6^ cfu/cm^2^) to a total dose of 6,360 J/m^2^, achieved a small (1.14) but significant log_10_ reduction in *C. difficile* 027 spore numbers. Exposing a less contaminated surface (10^5^ cfu/cm^2^) to the same UV-C dose achieved a 3 log_10_ reduction. The clumping of high numbers of spores may inhibit the penetration of UV-C rays. Spores within a clump may be shielded and protected by those directly exposed. Nonetheless, when dried onto a surface, high numbers of *C. difficile* NCTC 11209 spores (10^6^ cfu/cm^2^) were reduced by 3.55 log values within 60 seconds (i.e. after a comparatively lower dose of 3,180 J/m^2^; Table 3). Spores of wild type variants of *C. difficile* have also been shown to be more resistant to chemical disinfectants than those of laboratory strains [24]. Additionally, spore size can vary both within and between strains [25] and an increased spore diameter may reduce the ability of the UV-C rays to penetrate the various spore layers [26].

**Figure 4:**
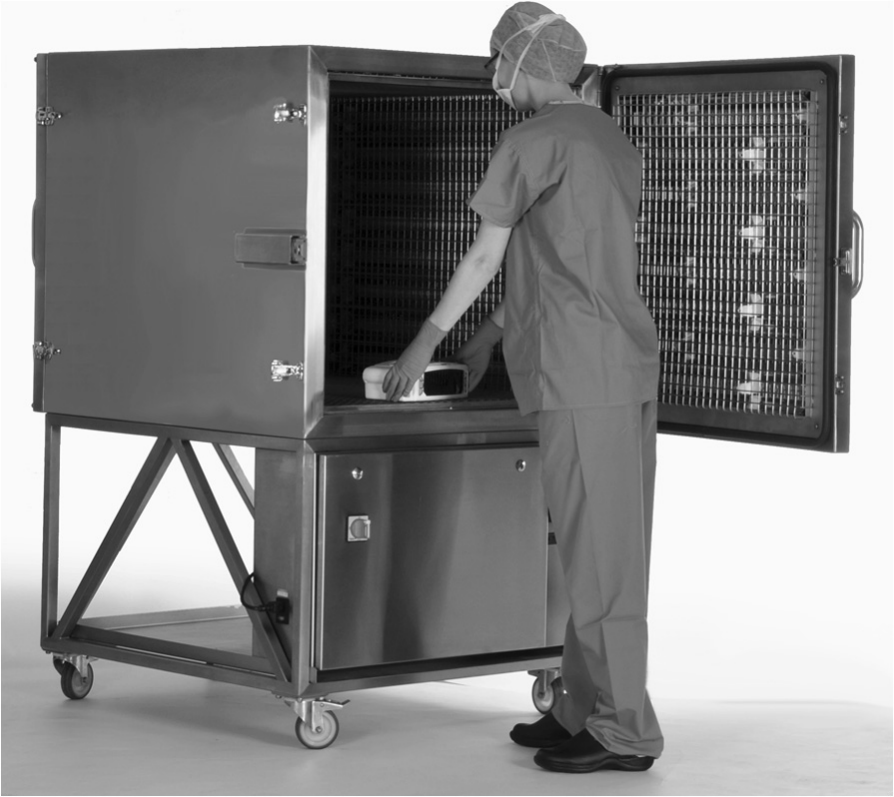
Placing an item to be disinfected inside the Nanoclave Cabinet.

There are no European Standard sporicidal surface tests for the medical area; current standards are suspension tests which require a 3 or 4 log_10_ reduction within 30, 60 or 120 minutes [27]. When used to disinfect a surface contaminated with *C. difficile* spores at levels equating to 10^3^ cfu/cm^2^, the Nanoclave Cabinet achieved a 3 log_10_ reduction within 2 minutes – a shorter, more relevant exposure time than those specified by current standards. However, the manufacturers of the Nanoclave stipulate that the Cabinet should only be loaded with one item at a time (as illustrated in Figure 4). Thus, in contrast to whole room decontamination devices, the total time required to disinfect a number of items using the Nanoclave Cabinet could be high, particularly if the cabinet is used for viral disinfection.

Adenovirus is associated with respiratory, ocular and gastrointestinal disease, especially in children. Once excreted, it can survive and remain infectious within the environment for up to 35 days. As a double stranded DNA virus, Adenovirus is particularly resistant to UV irradiation [28]. During the current study, the Nanoclave Cabinet rendered high levels of Adenovirus DNA (10^9^ viral genomes), on flat stainless steel sheets or ceramic tiles, undetectable by a sensitive PCR. However, to achieve this level of degradation (> 6 log_10_ reduction in detected viral DNA) it was necessary to expose the test surfaces to twelve 30-second UV cycles (i.e. a total dose of 19,080 J/m^2^). A lower exposure time may be required to achieve a 6 log_10_ reduction in viable virus as Adenovirus is likely to become non-viable before DNA becomes non-detectable by PCR.

It is also stated, both in the technical specifications document and the instructions for use of the Nanoclave Cabinet, that “*items to be disinfected must be physically clean before irradiation”.* Removal of visible soil is important both aesthetically and chemically. Organic soils are known to react with disinfectant molecules reducing their bioavailability. UV-C is also absorbed by organic materials [8] and whilst the Nanoclave Cabinet is not intended to be used to decontaminate heavily soiled invasive items, some non-critical patient care items may be difficult to manually clean. As with other UV-C irradiation devices [9], the efficacy of the Nanoclave Cabinet was not reduced by bovine serum albumin or red blood cells. However, it is acknowledged that the soiling experiments were only carried out using flat stainless steel discs and whilst the positioning of the discs within the Cabinet did not influence the reductions obtained, the presence of organic materials within recesses and/or areas of significant shadowing may effect the ability of the Nanoclave Cabinet to rapidly and effectively disinfect patient-care items.

## Conclusions

There are no standard test methods or acceptance requirements for equipment such as the Nanoclave Cabinet. During the current study, the test requirements for chemical disinfectants were used as the basis for the acceptance criteria. These stipulate that a bactericidal and sporicidal product should achieve a 5 log_10_- and a 3 log_10_ reduction respectively. The Nanoclave Cabinet effectively reduced the numbers of a range of potential pathogens including *Clostridium difficile* spores and Adenovirus from most, but not all, test surfaces and patient-care items. High level bacterial and viral disinfection (> 5 log_10_ reduction) was achieved within 1 and 6 minutes respectively suggesting that the Nanoclave Cabinet could be used to provide rapid and effective disinfection of patient-related equipment. However, bacteria did persist on some test sites; these areas may have been ‘in shadow’ due to individual item shape and other decontamination methods may be required. Furthermore, the Nanoclave Cabinet can only be loaded with one item at a time and the real life practicability of such a system was not assessed as part of this investigation. Laboratory studies do not necessarily replicate ‘in-use’ conditions and further studies are required to assess the acceptability and usability of the Nanoclave Cabinet within the clinical environment and its performance if Standard Operating Procedures are not adhered to.

## Competing interests

Nanoclave Technologies LLP (London, UK) provided temporary loan of the Nanoclave Cabinet and financial support to cover the costs of consumables. However, Nanoclave Technologies did not contribute to the study design nor the writing or editing of the manuscript.

## Authors’ contributions

SA, CB and MW carried out the bacterial investigations. EC-G carried out the Adenovirus study. GM drafted the manuscript. APRW conceived of the study. APRW, AF, CB and JH critically reviewed the manuscript. All authors participated in the design and co-ordination of the study and read and approved the final manuscript.

## Pre-publication history

The pre-publication history for this paper can be accessed here:

http://www.biomedcentral.com/1471-2334/12/174/prepub
